# Intranasal adenoviral neuraminidase vaccine induces local and systemic immunity and homosubtypic protection against influenza A viruses

**DOI:** 10.3389/fimmu.2026.1911139

**Published:** 2026-10-22

**Authors:** Wen-Chien Wang, Weiping Cao, Amrita Kumar, Wadzanai P. Mboko, Ram Kamal, Ekramy E. Sayedahmed, Suresh K. Mittal

**Affiliations:** 1 Department of Comparative Pathobiology, and Purdue Institute of Inflammation, Immunology and Infectious Disease, College of Veterinary Medicine, Purdue University, West Lafayette, IN, United States; 2 Influenza Division, Centers for Disease Control and Prevention, Atlanta, GA, United States

**Keywords:** bovine adenoviral vector, homosubtypic protection, human adenoviral vector, innate lymphoid cells (ILC), neuraminidase (NA), non-human adenoviral vector, T cell immune response

## Abstract

**Introduction:**

The rapid antigenic drift of influenza A hemagglutinin (HA) limits the effectiveness of current HA-focused vaccines. To address this challenge, we evaluated recombinant human (HAd) and bovine (BAd) adenoviral vectors expressing neuraminidase (NA) as an alternative strategy to elicit broad and protective mucosal immunity.

**Methods:**

BALB/c mice were immunized intranasally with HAd- or BAd-based vectors expressing N9 NA. Antigen-specific cellular and humoral immune responses were quantified locally and systemically. Immunized mice were subsequently monitored for survival and protective efficacy following lethal homosubtypic or heterosubtypic (Group 1 [H1N1] and Group 2 [H3N2]) influenza A viral challenges.

**Results:**

Intranasal immunization with both vectors induced strong systemic IgG and mucosal IgA responses, as well as robust systemic and local T-cell activation, providing complete protection against lethal homosubtypic challenge. Notably, the BAd-N9NA vector elicited stronger mucosal and cellular responses than the HAd-based counterpart. Regarding cross-protection, N9-based vaccination conferred partial protection against the Group 2 (H3N2) virus but failed against the Group 1 (H1N1) virus.

**Discussion:**

These findings demonstrate that NA-expressing adenoviral vectors induce potent mucosal and cellular immunity that mediates strong homosubtypic protection. While conserved structural epitopes exist across N1, N2, and N9 subtypes, heterosubtypic protection is limited and remains constrained by phylogenetic group lineages and sequence conservation. Furthermore, these results highlight the BAd platform as a highly potent delivery system for maximizing NA-specific immunogenicity, offering valuable insights for the design of broadly protective NA-based vaccines.

## 1 Introduction

Influenza A virus (IAV) continues to cause a substantial global health burden, driving seasonal epidemics and recurring zoonotic spillover events from avian influenza virus reservoirs including H5N1, H7N9, and H9N2 (1–3). The recent detection of avian influenza infections in cattle further raises concerns about an expanding range of hosts and cross-species susceptibility (4, 5). Current vaccination strategies largely target hemagglutinin (HA), aiming to induce neutralizing antibodies that block viral entry. However, HA undergoes frequent antigenic drift and occasional antigenic shift, creating recurrent mismatches between vaccine viruses and circulating viruses (6). These limitations underscore the need for next-generation vaccines, including those that target other viral antigens.

Neuraminidase (NA), the second major viral surface glycoprotein, is increasingly recognized as a promising complementary or alternative vaccine antigen (7). NA is less abundant on the virion, maintaining a typical HA: NA ratio of approximately 4:1 (8, 9). This structural imbalance contributes to immunodominance of HA and underrepresentation of NA-specific immunity (10, 11). Importantly, NA is more conserved across types and subtypes, and antibodies directed against NA can inhibit viral release, restrict spread, impede attachment through steric hindrance, and mediate Fc-dependent effector functions such as antibody-dependent cellular cytotoxicity (ADCC) and complement-dependent cytotoxicity (CDC) (12–16). Targeting NA through vaccination can therefore elicit protective mechanisms rarely achieved following natural infection or conventional HA-focused vaccination.

Multiple vaccine platforms, including inactivated viruses, recombinant proteins, and mRNA vaccines, have demonstrated that NA-specific immunity can provide meaningful cross-protection (17–19), with mucosal NA vaccination explicitly shown to enhance respiratory protection and reduce viral transmission (20, 21). However, generating such strong mucosal responses and robust cellular immunity remains a key challenge for NA-based vaccines. Recombinant adenoviral (Ad) vectors are well suited for this role, as they induce potent humoral and T-cell responses (22), and, when delivered intranasally, effectively stimulate mucosal IgA production and tissue-resident immunity in the respiratory tract (23). In addition to the established human Ad type 5 (HAd-5) vector (24), our laboratory has developed a bovine Ad type 3 (BAd-3) platform demonstrating enhanced immunogenicity in preclinical studies (25), providing a unique opportunity for side-by-side comparison of human and nonhuman Ad vectors.

NA proteins are phylogenetically divided into Group 1 (including N1) and Group 2 (including N2 and N9) (12). Given the persistent zoonotic and pandemic threats of H7N9, we selected N9 as a target to test whether it could elicit intra-group protection against N2 (seasonal H3N2 or zoonotic H9N2) or cross-group protection against N1 (seasonal H1N1 or zoonotic H5N1). Here, we generated recombinant HAd- and BAd-based vaccines expressing the NA gene from A/Shanghai/2/2013(H7N9). We evaluated their capacity to induce humoral and cellular immunity and assessed protection against homosubtypic and heterosubtypic viral challenge. Our results show that intranasal (i.n.) delivery of NA-expressing Ad vectors induces potent systemic and mucosal immunity and fully protects against lethal H7N9 infection. Furthermore, we demonstrate that the BAd-N9NA vector elicits stronger immune responses than its HAd counterpart, underscoring the value of alternative Ad platforms for next-generation influenza vaccine development.

## 2 Materials and methods

### 2.1 Cells and viruses

BHH/F5 (bovine–human hybrid cells expressing Ad E1 proteins) (23), BHH/F5-I-SceI cells (BHH/F5 cells expressing the I-SceI endonuclease) (23), BHH2C bovine–human hybrid clone 2C cells (26), human embryonic kidney HEK293 cells (CRL-1573, ATCC, Manassas, VA, USA), 293Cre cells expressing Cre recombinase (27), and Madin–Darby canine kidney (MDCK) cells (CCL-34, ATCC) were maintained in Dulbecco’s Modified Eagle Medium (DMEM; Corning, Corning, NY, USA) supplemented with 10% fetal clone III serum (FCIII; Hyclone, Logan, UT, USA) and antibiotics. All cells were cultured at 37 °C with 5% CO_2_.

Challenge influenza viruses including A/PR/8/34(H1N1), A/HK/1/68(H3N2), A/Anhui/1/2013(H7N9), and A/Shanghai/2/2013(PR8-H7N9) were propagated in 10-day-old specific pathogen-free (SPF) embryonated chicken eggs (AVS Bio, Norwich, CT, USA) for 72 h at 33 °C. Allantoic fluid was harvested, clarified, aliquoted, and stored at −80 °C. Viral titers were determined using a 50% tissue culture infectious dose (TCID_50_) assay in MDCK cells.

The A/Shanghai/2/2013(PR8-H7N9) (PR8-IDCDC-RG32A) virus is a 6:2 reassortant strain containing the HA and NA genes from A/Shanghai/2/2013(H7N9) and the six internal genes (PB2, PB1, PA, NP, M, and NS) from the mouse-adapted A/Puerto Rico/8/1934(H1N1) backbone (28).

### 2.2 Generation of adenoviral vectors

NA sequences from A/Shanghai/2/2013(H7N9) (YP_009118481), A/PR/8/34(H1N1) (BDD17680), and A/HK/1/68(H3N2) (AAO46469), and the HA sequence from A/Shanghai/2/2013(H7N9) (YP_009118475) were retrieved from NCBI GenBank. Codon-optimized gene sequences (rodent-optimized; Genscript, Piscataway, NJ, USA) were synthesized and cloned into expression cassettes flanked by the human cytomegalovirus (HCMV) promoter and bovine growth hormone (BGH) polyadenylation signal.

#### 2.2.1 HAd vector production

HAd vectors were generated via Cre-recombinase-mediated site-specific recombination in 293Cre cells as previously described (27). Vectors produced included HAd-N9NA, HAd-N1NA, HAd-N2NA, and HAd-H7HA. Viral particles were purified using cesium chloride (CsCl) density-gradient ultracentrifugation and titrated in BHH2C cells (26) via plaque assay.

#### 2.2.2 BAd vector production

BAd vectors were produced by I-SceI-mediated genome release following transfection into BHH/F5-I-SceI cells as previously described (23). Vectors generated included BAd-N9NA and BAd-H7HA, along with BAd-ΔE1E3 (E1/E3-deleted empty control). Purification was performed by CsCl ultracentrifugation, and titers were determined in BHH/F5-I-SceI cells (23) via plaque assay.

### 2.3 Western blot analysis

To verify transgene expression, HEK293 or BHH/F5 cells were infected with HAd or BAd vectors at an MOI of 2 PFU/cell. At 48 h post-infection, cells were harvested and lysed using established protocols (23). To preserve the oligomeric conformation of the NA protein, lysates were resolved under non-boiled conditions using an SDS-PAGE system without thermal denaturation. Membranes were blocked and incubated with a mouse anti-H7N9 NA monoclonal antibody (1:2,000; Kerafast, Newark, CA, USA), followed by an HRP-conjugated goat anti-mouse secondary antibody (1:10,000; Thermo Fisher Scientific, Waltham, MA, USA). A pre-conjugated β-actin HRP antibody (1:5,000; Thermo Fisher Scientific) was used as a loading control. Signals were developed with SuperSignal West Pico PLUS substrate (Thermo Fisher Scientific) and captured on an Azure 280 imaging system (Azure Biosystems).

### 2.4 Animal experiments

All animal studies conducted in BSL2 or BSL3 containment at CDC, including enhancements as required by the US Department of Agriculture, were approved by the Institutional Biosafety Committee (IBC; file no. 2017.106) and Institutional Animal Care and Use Committee (IACUC; protocols 2930GANMOUC and 2759YORMOUC) Animal studies conducted in an ABSL-2^+^ facility at Purdue University were approved by IBC (file no. 08-0150-24) and IACUC (protocol no. 1201000580). Female mice were utilized exclusively in this study to prevent aggressive cage-mate fighting and potential injury-associated cross-infections, which can confound long-term body weight and survival evaluations. The exclusive use of a single sex is acknowledged as a limitation regarding generalizability across sexes.

#### 2.4.1 HAd Immunogenicity and homosubtypic challenge

Six to eight-week-old female BALB/c mice (n = 15 per group; Jackson Laboratory, Bar Harbor, ME, USA) were anesthetized with intraperitoneal (i.p.) injection of ketamine (100 mg/kg) and xylazine (10 mg/kg), and intranasally (i.n.) administered 20 µL of Ad vaccines. Mice received 3 × 10^7^ PFU of HAd-ΔE1E3, HAd-N9NA, or HAd-H7HA following a prime–boost schedule at a 4-week interval.

Blood was collected three weeks after both immunizations. Subsequently, mice were humanely euthanized via an i.p. injection of an overdose of ketamine (200 mg/kg) and xylazine (20 mg/kg). Death was confirmed by bilateral thoracotomy and heart removal. Bone marrow, lungs, spleens, and mediastinal lymph nodes (MLN) were then collected three weeks post-boost for immunological analyses.

A separate cohort was challenged i.n. with 5 mouse lethal dose 50 (MLD_50_) of A/Anhui/1/2013(H7N9) four weeks after boosting in an ABSL-3 facility. Body weight and survival were monitored for 14 days. At the experimental endpoint or upon exceeding 25% weight loss, mice were humanely euthanized via an i.p. overdose of ketamine/xylazine, verified by cervical dislocation.

#### 2.4.2 HAd heterosubtypic challenge

BALB/c mice (n = 5 per group) received 3 × 10^7^ PFU of HAd-ΔE1E3, HAd-N1NA, HAd-N2NA, HAd-N9NA, or PBS via the i.n. route using the same prime–boost schedule. Positive controls were immunized i.p. with 200 HA units of A/PR/8/34(H1N1) or A/HK/1/68(H3N2).

Four weeks post-boost, mice were challenged with either 10 MLD_50_ of A/PR/8/34(H1N1) or 2 MLD_50_ of A/HK/1/68(H3N2). Survival and weight loss were monitored for 17 days. At the experimental endpoint or upon reaching humane endpoints (weight loss >25%), mice were humanely euthanized via an i.p. overdose of ketamine/xylazine, verified by cervical dislocation.

#### 2.4.3 BAd immunogenicity and homosubtypic challenge

Eight-week-old female BALB/c mice (n = 5 per group) received 3 × 10^7^ PFU of HAd-ΔE1E3, BAd-ΔE1E3, HAd-N9NA, BAd-N9NA, or BAd-H7HA via the i.n. route; PBS served as a negative control. All groups followed a prime–boost regimen with a 4-week interval.

Four weeks after boosting, mice were humanely euthanized via an i.p. overdose of ketamine/xylazine, and death was confirmed by heart removal. Serum, lung washes, and spleens were collected for immunological evaluation. In a separate cohort, mice were challenged with 10 MLD_50_ of A/PR/8/34(H1N1), A/HK/1/68(H3N2), or A/Shanghai/2/2013(PR8-H7N9). Morbidity and mortality were monitored for 14 days. At the experimental endpoint or upon reaching humane endpoints (weight loss >25%), mice were humanely euthanized via an i.p. overdose of ketamine/xylazine, verified by cervical dislocation.

### 2.5 ELISpot assay

ELISpot assays were performed as described previously (29).

#### 2.5.1 B-cell ELISpot

Multiscreen plates were coated with UV-inactivated PR8-H7N9 virus or recombinant NA protein [A/Shanghai/1/2013(H7N9), BEI Resources, NR-44080]. For virus inactivation by UV irradiation, viruses were placed on ice and irradiated using a 254-nm UV stratalinker (Stratagene, La Jolla, CA) for 30 min. Bone marrow cells or splenocytes were incubated for 48 h. Secreted antibodies were detected using biotin-conjugated anti-mouse IgA or IgG (Mabtech, Cincinnati, OH, USA), followed by streptavidin–alkaline phosphatase (ALP) and BCIP/NBT substrate. Spots were quantified using an AID iSpot analyzer.

#### 2.5.2 T-cell ELISpot

Plates were coated with anti-mouse IFN-γ or IL-2 capture antibodies (Mabtech). Splenocytes were stimulated for 48 h with inactivated A/Shanghai/2/2013(H7N9). Detection used biotin-labeled antibodies and streptavidin-ALP, followed by BCIP/NBT substrate. Spots were quantified using an AID iSpot analyzer.

### 2.6 Viral neutralization assay

Virus neutralization (VN) was performed as described previously (30). Heat-inactivated sera were serially diluted two-fold and incubated with 100 TCID_50_ of A/Shanghai/2/2013 (H7N9) for 1 h at 37 °C. Mixtures were added to MDCK monolayers and incubated for 48 h. Hemagglutination (HA) was assessed using 0.5% turkey red blood cells (RBCs). VN titers were defined as the reciprocal of the highest dilution yielding complete inhibition.

### 2.7 ELISA

ELISA procedures followed previously described methods (23). Plates were coated with UV-inactivated H7N9 virus or recombinant NA protein (1 µg/mL; BEI Resources) overnight at 4 °C. After blocking, serum or lung wash samples were serially diluted (2-fold for HAd studies; 10-fold for BAd studies) starting at 1:10.

HRP-conjugated anti-mouse IgG, IgG_1_, IgG_2a_, or IgA (Invitrogen, Waltham, MA, USA) were applied. Plates were developed with TMB and stopped with 2 N H_2_SO_4_. Absorbance was measured at 450 nm. Endpoint titers were defined as the highest dilution exceeding the blank mean + 2 SD. Endpoint titers were defined as the highest serial dilution yielding an optical density (OD_450_) value that exceeded a manual cutoff threshold, calculated as the mean OD_450_ of blank wells plus two standard deviations (mean + 2 SD). The limit of detection (LOD) for both studies was an endpoint titer of 10. For statistical analysis and graphical presentation, samples with titers below the LOD were assigned a value of 5 (half of the baseline dilution). Data from HAd immunogenicity studies were expressed after log_2_ transformation, whereas data from BAd immunogenicity studies were expressed after log_10_ transformation.

### 2.8 Flow cytometry

Flow cytometry was performed as described previously (31) with modifications. Splenocytes, MLN cells, or lung cells (5 × 10^6^ for splenocytes, 2×10^6^ for MLN and lung cells) in complete Roswell Park Memorial Institute 1640 (RPMI-1640) medium (Corning) were stimulated with or without inactivated A/Shanghai/2/2013(H7N9) in the presence of protein transport inhibitors (Invitrogen) for 6 h.

Following stimulation, cells underwent washing with fluorescence-activated cell sorting (FACS) buffer (comprising 1% FBS in PBS) and subsequent 15-min staining at 4 °C in the dark using the LIVE/DEAD Fixable Near-IR Dead Cell Stain Kit (Invitrogen, L34975). To block Fc receptors, samples were incubated with a rat anti-mouse CD16/CD32 antibody (clone 93; Invitrogen, 14-0161-82) for 15 min at 4 °C, followed by a single wash with FACS buffer. Surface antigen labeling was performed by incubating the cells with specific monoclonal antibodies (mAbs) including anti-CD3 FITC (clone 17A2; Invitrogen, 11-0032-82), anti-CD4 PE-Cy7 (clone RM4-5; Invitrogen, 25-0042-82), and anti-CD8 APC (clone 53-6.7; Invitrogen, 17-0081-82) for 30 min at 4 °C. Next, the cells were washed twice with FACS buffer prior to fixation and permeabilization using the Cytofix/Cytoperm Kit (BD Biosciences, Franklin Lakes, NJ, USA). After a single rinse with permeabilization buffer, intracellular cytokines were stained with anti-IFN-γ PE (clone XMG1.2; Invitrogen, 12-7311-82), anti-IL-2 BV421 (clone 5344.111; BD Biosciences, 562914), and anti-TNF-α PerCP-Cy5.5 (clone MAB11; BD Biosciences, 560679) for 30 min at 4 °C in the dark. Finally, the cells were washed twice and resuspended in FACS buffer. Data acquisition was executed on an FACSymphony A1 cytometer (BD Biosciences), and subsequent data analysis was performed using FlowJo v10 software. Gating thresholds for cytokine-positive populations were strictly determined using the unstimulated control cells (cells cultured in medium alone without viral stimulation) to account for background fluorescence. The complete gating strategy is shown in **Supplementary Figure 1**.

### 2.9 Statistical analysis

Mice were randomly assigned to different experimental and control groups upon arrival. Body weight, clinical signs, and survival were monitored daily following viral challenge. For sample processing and immunological assays, samples were processed and analyzed using standardized, objective gating and automated counting software to minimize potential investigator bias.

Data are presented as mean ± SD or individual values. Details of sample size (n), statistical tests, and comparison groups are provided in figure legends. In detail, to account for the longitudinal nature of body weight changes, data were analyzed using a Two-way repeated-measures ANOVA, or a mixed-effects model in the presence of missing values from animal mortality. Both models were followed by Dunnett’s *post-hoc* test for point-by-point comparisons. Time points following total group mortality were excluded to avoid baseline artifact. Survival curves were evaluated using the global Log-rank (Mantel-Cox) test, with subsequent pairwise log-rank comparisons to identify specific treatment effects against controls. ELISA and neutralization titers were log_10_ transformed prior to parametric analysis. Significance was defined as p < 0.05. Analyses were performed using GraphPad Prism 10 (GraphPad Software, San Diego, CA, USA).

## 3 Results

### 3.1 HAd-N9NA induces strong humoral immunity

To generate HAd vectors (HAd-N9NA, HAd-N1NA, HAd-N2NA, and HAd-H7HA), we constructed expression cassettes encoding NA from A/Shanghai/2/2013(H7N9), A/PR/8/34(H1N1), or A/HK/1/68(H3N2), and HA from A/Shanghai/2/2013(H7N9) (**Figure 1A**). Recombinant vectors were produced using Cre-mediated recombination, and protein expression was validated by immunoblotting. Under non-boiled conditions in the presence of SDS, cells infected with HAd-N9NA revealed a prominent high-molecular-weight band at approximately 240 kDa corresponding to the NA oligomer, accompanied by a faint but distinct band near 60 kDa representing the monomeric subunit. (**Figure 1A**). To establish a traditional benchmark for comparison, the HA-expressing Ad vector (HAd-H7HA) was included alongside NA-expressing constructs to evaluate their respective immunological profiles within the same Ad platform. Additionally, the functional expression of the HAd-H7HA vector has been previously verified and well-characterized (33). The immunization and challenge schedule is shown in **Figure 1B**. BALB/c mice were immunized i.n. with 3×10^7^ PFU of each vector on a prime-boost regimen. Serum, bone marrow, and spleen were collected to quantify B-cell responses by ELISA and ELISpot.

**Figure 1:**
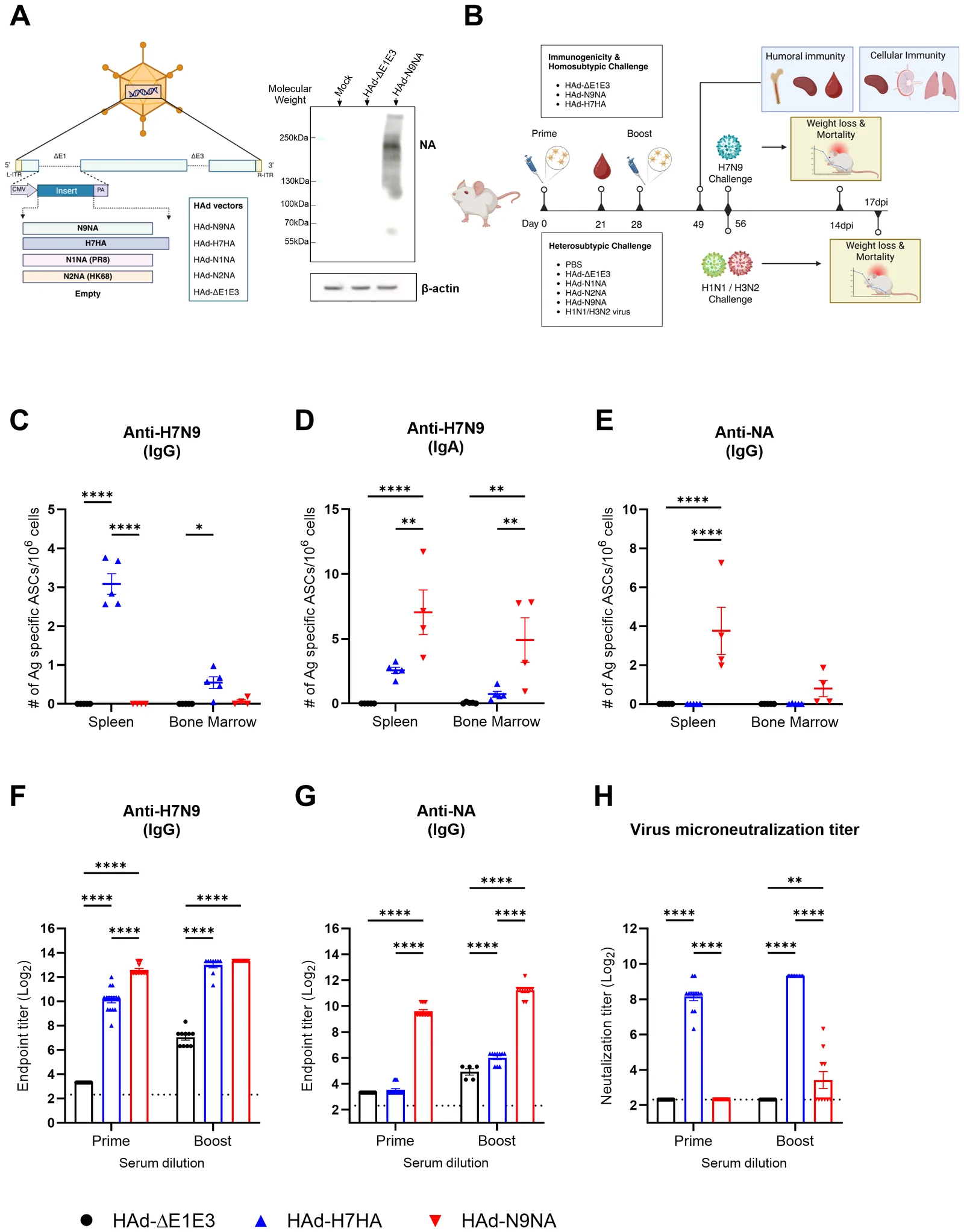
Characterization of human adenoviral (HAd) vector constructs, experimental study design, and vaccine-induced humoral immune responses. **(A)** Schematic representation of HAd-vector expression cassettes containing neuraminidase (NA) genes from H1N1, H3N2, or H7N9, or the hemagglutinin (HA) gene from H7N9 (left). Expression of HAd-N9NA was confirmed by immunoblot analysis with β-actin used as a loading control (right). CMV, cytomegalovirus promoter; PA, bovine growth hormone (BGH) polyadenylation signal; ITR, inverted terminal repeats. **(B)** Experimental study design for immunogenicity and challenge studies using a prime-boost regimen. BALB/c mice were immunized intranasally (i.n.) with HAd vectors and received a homologous boost four weeks post-primary immunization. **(C–E)** B-cell responses in the spleen and bone marrow (n=5) were assessed four weeks post-boost by ELISpot assay to detect antibody-secreting cells (ASCs) for **(C)** anti-H7N9 IgG, **(D)** anti-H7N9 IgA, and **(E)** anti-NA IgG. **(F–H)** Humoral responses in serum (n=5-15) collected three weeks post-prime and boost. Antibody titers were determined by ELISA for **(F)** anti-H7N9 IgG and **(G)** anti-NA IgG, and by **(H)** microneutralization assay. ELISA data are presented as endpoint titers; the cutoff was defined as the mean of blank wells plus two standard deviations (SD). Dotted lines indicate the limit of detection (LOD = log_2_10). Each symbol represents an individual animal, with error bars indicating the SD. Statistical significance was determined by one-way ANOVA followed by Dunnett’s *post hoc* test relative to the ΔE1E3 control group (*p ≤ 0.05; **p ≤ 0.01; ****p ≤ 0.0001).

As expected, HAd-H7HA induced the highest number of H7N9-specific IgG antibody-secreting cells (ASCs) (**Figure 1C**). In contrast, HAd-N9NA generated significantly higher IgA responses (**Figure 1D**) and more NA-specific IgG ASCs (**Figure 1E**). Both groups exhibited elevated H7N9-binding IgG following boosting, demonstrating that both HA and NA antigens are highly immunogenic in this platform. Consistent with the mechanistic roles of each antigen, HA immunization produced higher neutralizing titers, while NA immunization exhibited a detectable replication-limiting activity within the assay (**Figures 1F–H**). These findings indicate that HA-mediated neutralization remains the dominant mechanism for blocking viral entry, while NA-directed immunity appears to offer complementary, replication-limiting activity *in vitro*.

### 3.2 HAd-N9NA vaccination induces potent cellular immune responses

Three weeks after the booster immunization, cells were isolated from the spleen, MLN, and lungs to assess H7N9-specific cellular immunity. Flow cytometry was performed to quantify IFN-γ, IL-2, and TNF-α production in CD4^+^ and CD8^+^ T cells following stimulation with H7N9 virus.

HAd-H7HA vaccine induced significantly higher frequencies of cytokine-positive CD4^+^ T cells in the spleen compared with unstimulated controls, and HAd-N9NA elicited comparable higher level (**Figure 2A**), demonstrating a strong systemic response. Consistent increases in CD4^+^ T-cell activity were also detected in the MLN and lungs (**Figures 2C, E**), indicating effective induction of local cellular immunity.

**Figure 2:**
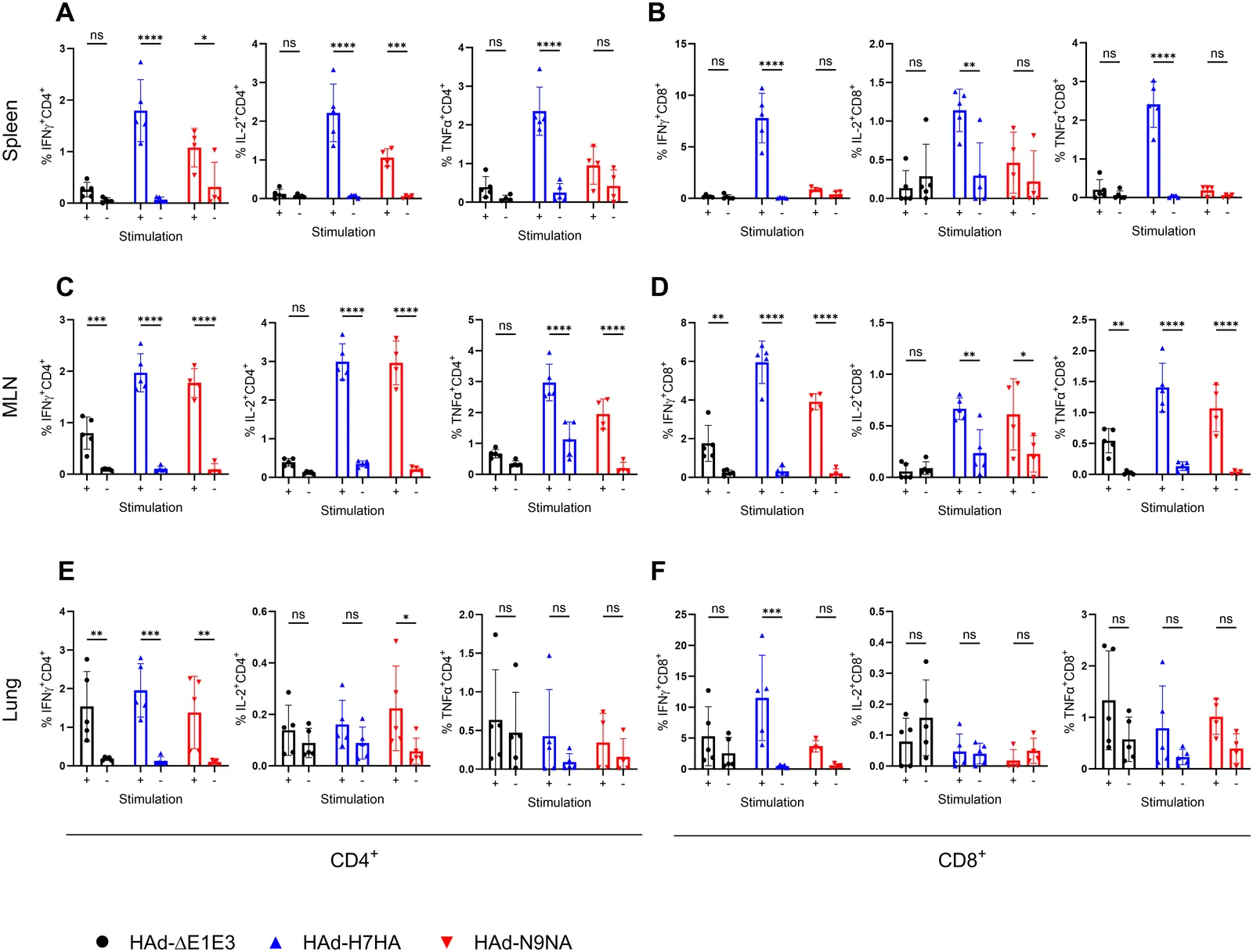
Vaccine-induced cellular immune responses in HAd-vector-immunized BALB/c mice. Three weeks post-boost, cells were isolated from the spleen, mediastinal lymph nodes (MLN), and lungs of immunized mice (n=5). Isolated cells were either stimulated (+) or not stimulated (–) with H7N9 influenza virus, and cytokine expression in CD4^+^ or CD8^+^ T cells was quantified by flow cytometry. Percentages of CD4^+^ or CD8^+^ T cells expressing IFN-γ, IL-2, or TNF-α in the **(A, B)** spleen, **(C, D)** MLN, and **(E, F)** lungs are shown. Each symbol represents an individual animal, and error bars indicate the standard deviation (SD). Statistical significance was determined by one-way ANOVA followed by Dunnett’s *post-hoc* test relative to the ΔE1E3 control group (ns: non-significant; *p ≤ 0.05; **p ≤ 0.01; ***p ≤ 0.001; ****p ≤ 0.0001).

In contrast, CD8^+^ T-cell activation differed between vaccine groups. The HAd-H7HA vaccine elicited markedly elevated CD8^+^ cytokine responses in both systemic and local tissues (**Figures 2B, D, F**). The HAd-N9NA group showed enhanced CD8^+^ T-cell activation only in the MLN (**Figure 2D**), with minimal responses in the spleen and lungs (**Figures 2B, F**).

Notably, low-level cytokine expression was observed in the HAd-ΔE1E3 control group. This finding is consistent with previous observations that vector-induced innate lymphoid cell (ILC) activity can promote nonspecific T-cell stimulation (34).

In summary, HAd-N9NA vaccination induces strong systemic and local H7N9-specific CD4^+^ T-cell responses, whereas HAd-H7HA generates robust activation of both CD4^+^ and CD8^+^ T-cell populations.

### 3.3 HAd-N9NA provides complete homosubtypic protection but limited heterosubtypic protection

To assess vaccine efficacy, mice were challenged four weeks post-boost with a lethal dose of A/Anhui/1/2013(H7N9). Both HAd-N9NA and HAd-H7HA conferred complete protection, preventing weight loss and mortality. Notably, 60% of the HAd‑ΔE1E3 empty-vector control group also survived the infection, with only succumbing (**Figure 3**), suggesting a substantial contribution from vector-induced innate or non-specific protective effects. These data confirm that i.n. delivery of NA using an Ad vector is sufficient to induce full homosubtypic protection against H7N9, while highlighting the inherent, non-specific protection provided by the vector backbone.

**Figure 3:**
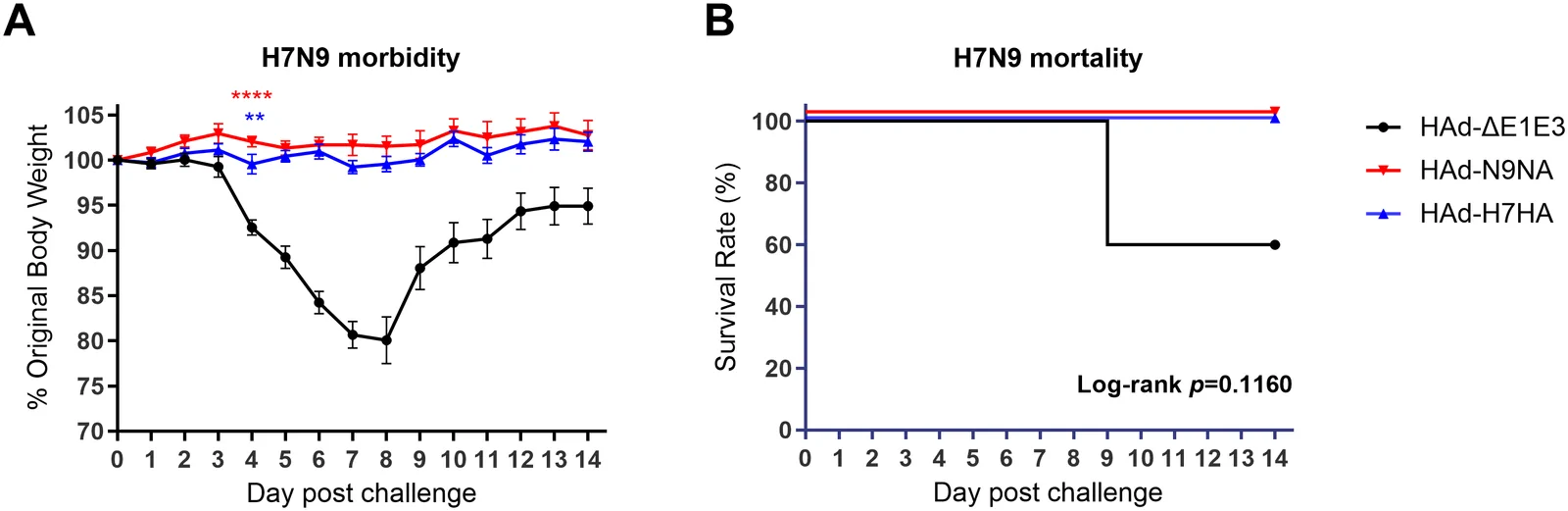
Morbidity and mortality in HAd-vector-immunized BALB/c mice following lethal challenge with H7N9 influenza virus. BALB/c mice (n=5) were immunized intranasally (i.n.) with HAd-ΔE1E3, HAd-N9NA, or HAd-H7HA. Four weeks post-primary immunization, mice received a homologous boost. At four weeks post-boost, mice were challenged with 5 mouse lethal dose 50 (MLD_50_) of A/Anhui/1/2013(H7N9) and monitored for **(A)** morbidity (percentage of original body weight) and **(B)** mortality (survival rate) for 14 days post-challenge. Weight longitudinal data were analyzed using a two-way repeated-measures ANOVA or a mixed-effects model followed by Dunnett’s *post-hoc* test. The asterisk indicates the onset of significant body weight divergence between the vaccinated group and the PBS control group, which remained statistically significant throughout the remainder of the observation period. Statistical analysis of the survival curves was performed using the global log-rank (Mantel-Cox) test. Individual *p*-values indicated on the graph represent the specific statistical significance compared to the HAd-ΔE1E3 control group using pairwise log-rank tests. (**p ≤ 0.01; ****p ≤ 0.0001).

To assess heterosubtypic cross-protection. Mice were vaccinated with HAd-N9NA, HAd-N1NA, or HAd-N2NA and challenged with A/PR/8/34 (H1N1; Group 1) or A/HK/1/68 (H3N2; Group 2). Sequence identities between vaccine and challenge NA proteins are summarized in **Supplementary Table 1**.

As expected, HAd-N1NA completely protected against homologous H1N1 challenge, whereas all other groups, including HAd-N9NA, exhibited 100% mortality (**Figures 4A, B**). Against H3N2, HAd-N2NA fully protected mice, while HAd-N9NA conferred partial (60%) survival (**Figures 4C, D**). Taken together, these results show that NA-based Ad vaccines provide strong subtype-matched (homosubtypic) immunity, but the heterosubtypic protection is limited and is tightly linked to phylogenetic group lineages and epitope-specific sequence conservation rather than broad structural similarities.

**Figure 4:**
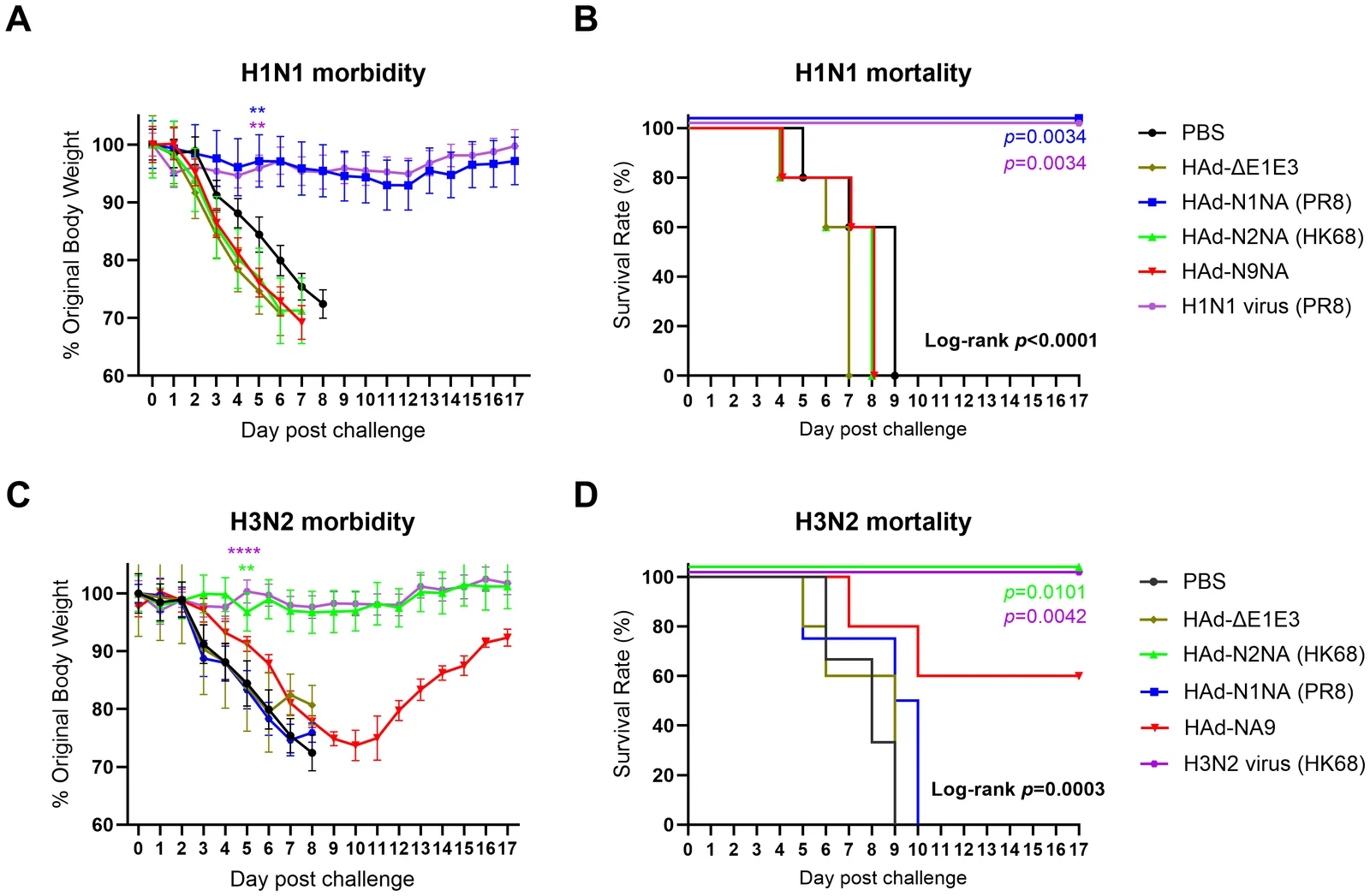
Protection efficacy against lethal H1N1 or H3N2 infection in HAd-vector-vaccinated mice. To evaluate the heterosubtypic protection, BALB/c mice (n=5) were immunized intranasally (i.n.) with PBS, HAd-ΔE1E3, HAd-N1NA, HAd-N2NA, or HAd-N9NA. Positive control groups were immunized intraperitoneally (i.p.) with 200 HA units of A/PR/8/34(H1N1) or A/HK/1/68(H3N2). 4 weeks after a booster dose, mice were challenged with 10 mouse lethal dose 50 (MLD_50_) of H1N1 **(A, B)** or 2 MLD_50_ of H3N2 **(C, D)** and monitored for morbidity and mortality for 17 days. Weight longitudinal data were analyzed using a two-way repeated-measures ANOVA or a mixed-effects model followed by Dunnett's post-hoc test. The asterisk indicates the onset of significant body weight divergence between the vaccinated group and the PBS control group, which remained statistically significant throughout the remainder of the observation period. Statistical analysis of the survival curves was performed using the global log-rank (Mantel-Cox) test. Individual *p-values* indicated on the graph represent the specific statistical significance compared to the PBS control group using pairwise log-rank tests. (**, p ≤ 0.01; ***, p ≤ 0.001; ****, p ≤ 0.0001).

### 3.4 BAd-N9NA elicits enhanced humoral and cellular immunity compared to HAd-N9NA

We next explored whether nonhuman Ad vectors (BAd) could improve NA-specific humoral immunity and H7N9 virus-specific cellular immunity. Building on our previous work demonstrating that BAd vectors induce stronger innate and adaptive responses than HAd vectors (25), we constructed BAd-N9NA and BAd-H7HA vectors (**Figure 5A**). Similar to the HAd construct, immunoblot analysis confirmed the robust expression of BAd-N9NA, characterized by the typical oligomeric smear and monomeric band (**Figure 5A**). We then compared them head-to-head with their HAd counterparts using the same prime-boost i.n. regimen.

**Figure 5:**
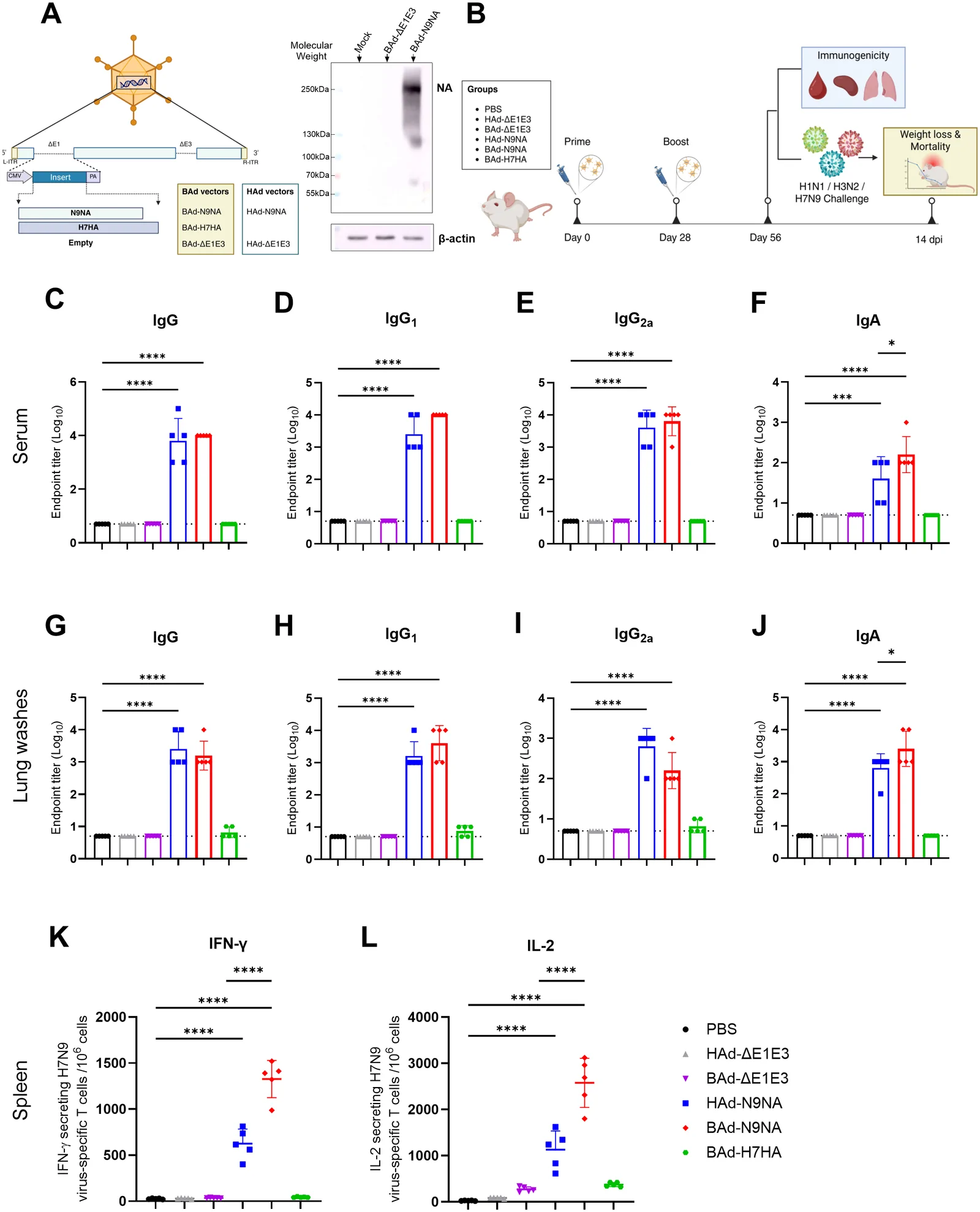
Human adenoviral (HAd) and bovine adenoviral (BAd) vector constructs, experimental study design, and development of immune responses in immunized mice. **(A)** Schematic representation of expression cassettes containing the NA or HA genes from H7N9 influenza virus used to generate HAd and BAd vectors (left). Expression of BAd-N9NA was confirmed by immunoblot analysis with β-actin used as a loading control (right). CMV: cytomegalovirus promoter; PA: bovine growth hormone (BGH) polyadenylation signal; ITR: inverted terminal repeats. **(B)** Experimental study design for immunogenicity and challenge studies using HAd or BAd vector vaccines. **(C–J)** Vaccine-induced humoral immune responses. Sera and lung washes (n=5) were collected four weeks post-boost. **(C–F)** Serum and **(G–J)** lung washes were analyzed by ELISA for NA-specific IgG, IgG_1_, IgG_2a_, and IgA titers. **(K–L)** Vaccine-induced cellular immune responses. Splenocytes (n=5) were collected four weeks post-boost and stimulated with H7N9 virus. The number of H7N9 virus-specific **(K)** IFN-γ or **(L)** IL-2-secreting T cells was determined by ELISpot assay. ELISA cutoffs were defined as the mean of blank wells plus two standard deviations (SD). Dotted lines indicate the limit of detection (LOD = log_10_5). Each symbol represents an individual animal, and error bars indicate SD. Data were analyzed by one-way ANOVA followed by Tukey’s *post hoc* test for pairwise comparisons among all experimental groups; significant differences between specific groups are indicated by asterisks in the panels (*p ≤ 0.05; ***p ≤ 0.001; ****p ≤ 0.0001).

Both vectors induced strong systemic (IgG, IgG_1_, IgG_2a_) and mucosal (IgA) antibodies in serum and lung washes (**Figures 5C–J**). However, BAd-N9NA elicited higher mucosal IgA titers, suggesting enhanced efficacy in driving local mucosal immunity.

To further assess T-cell responses, we performed ELISpot analysis of splenocytes stimulated with H7N9 virus. Both BAd-N9NA and HAd-N9NA produced significantly higher frequencies of IFN-γ and IL-2-secreting cells compared with PBS controls. Notably, BAd-N9NA elicited even higher frequencies than its HAd counterpart (**Figures 5K, L**), confirming its enhanced immunogenicity.

Despite these stronger immune responses, BAd-N9NA conferred the same pattern of protection observed with HAd-N9NA, which showed complete protection against H7N9, partial protection against H3N2, and no protection against H1N1 (**Figure 6**). These results further support that NA-based cross-protection is primarily dictated by sequence homology rather than vector potency or immune response magnitude.

**Figure 6:**
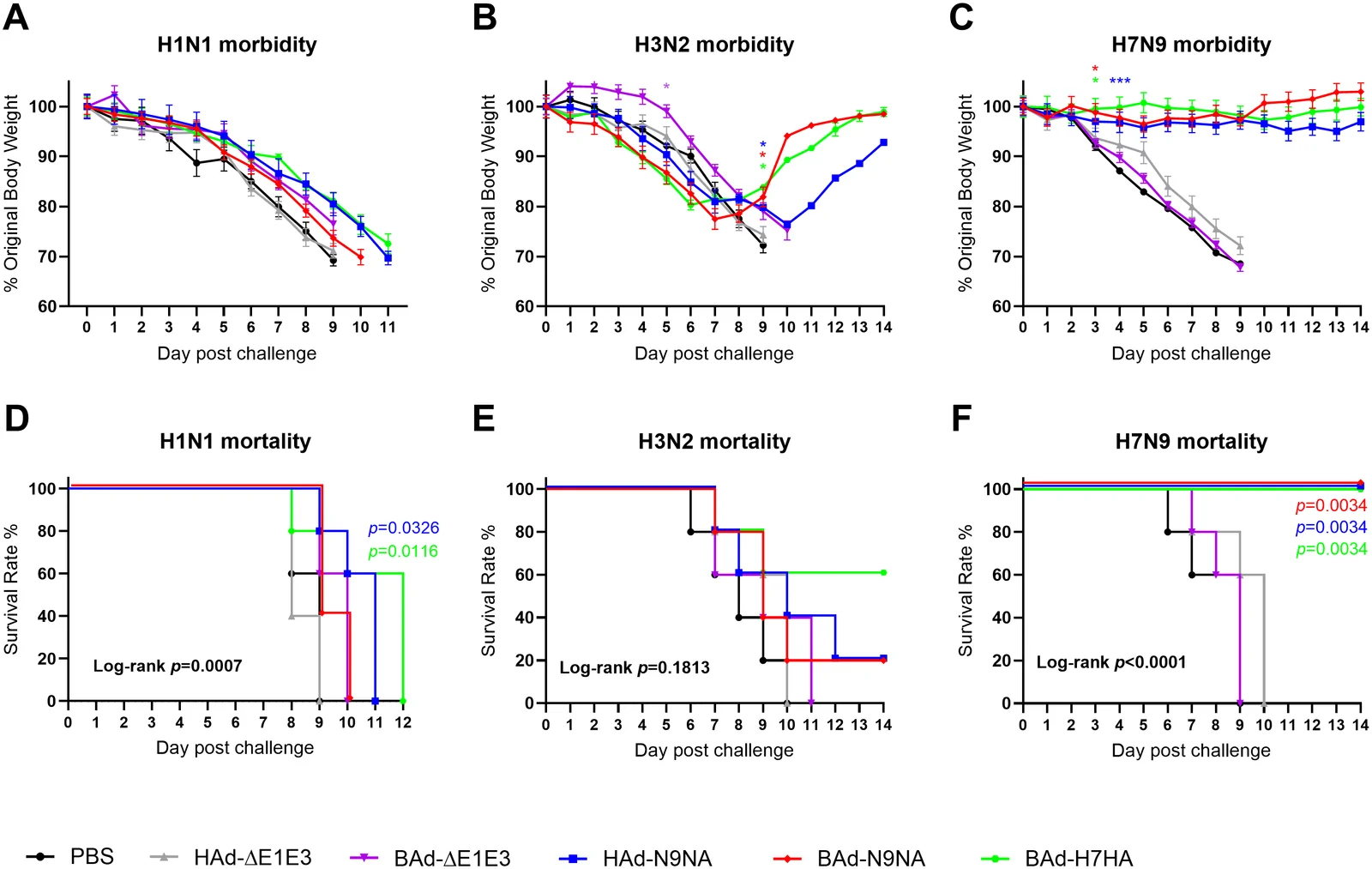
Protective efficacy of HAd or BAd-vector vaccines against lethal H1N1, H3N2, or H7N9 challenges. BALB/c mice (n=5) were immunized intranasally (i.n.) with PBS, HAd-ΔE1E3, BAd-ΔE1E3, HAd-N9NA, BAd-N9NA, or BAd-H7HA. Four weeks post-boost, mice were challenged with 10 mouse lethal dose 50 (MLD_50_) of A/PR/8/34(H1N1) **(A, D)**, A/HK/1/68(H3N2) **(B, E)**, or A/Shanghai/2/2013(PR8-H7N9) **(C, F)**. Morbidity (weight loss) and mortality (survival) were monitored daily for 14 days post-challenge. Weight data were analyzed using a two-way repeated-measures ANOVA or a mixed-effects model followed by Dunnett’s *post-hoc* test. The asterisk indicates the onset of significant body weight divergence between the vaccinated group and the PBS control group, which remained statistically significant throughout the remainder of the observation period. Statistical analysis of the survival curves was performed using the global log-rank (Mantel-Cox) test. Individual *p*-values indicated on the graph represent the specific statistical significance compared to the PBS control group using pairwise log-rank tests. (*p ≤ 0.05; ***p ≤ 0.001).

## 4 Discussion

The continual evolution of influenza viruses and the potential emergence of pandemic and zoonotic virus variations highlight the limitations of current HA-based vaccines. Although HA-specific antibodies provide strong neutralizing activity, their effectiveness is often undermined by rapid antigenic drift (35). To address this challenge, numerous studies have explored immune responses targeting more conserved viral components such as chimeric HA, the HA2 stem, the ectodomain of the matrix protein 2 (M2e), and nucleoprotein (NP) as strategies for developing broadly protective influenza vaccines (23, 36–38). In this study, we focused on NA as an alternative vaccine antigen due to its relatively lower sequence variability and potential to confer broader cross-protection. By employing Ad delivery systems, we aimed to leverage both mucosal and cellular immunity to overcome the limitations of conventional seasonal influenza vaccination.

Our analysis of the humoral immune responses revealed distinct antibody profiles in spleen and bone marrow between the HA- and NA-targeted vaccines. HAd-H7HA induced higher IgG titers, whereas HAd-N9NA elicited substantially stronger IgA responses. This divergence in antibody isotypes likely reflects the intrinsic immunogenic differences between the two glycoproteins when antigenic competition is removed through separate Ad-based delivery (39). Despite these differences in isotype distribution, both vaccines generated high and comparable levels of H7N9-specific IgG after boosting, indicating that the overall magnitude of virus-binding antibodies is similar in both groups and confirming that both HA and NA are highly immunogenic when expressed from Ad vectors. As anticipated, HA-specific antibodies displayed significantly higher neutralization titers due to their primary role in blocking viral entry (40). Notably, however, the HAd-N9NA group exhibited a marked increase in inhibitory activity in the microneutralization assay following the booster dose. This effect is likely mediated by NA-specific antibodies restricting multi-cycle viral replication and virion propagation within the culture (41). Such replication-limiting activity *in vitro* can consequently manifest as a neutralization-like reduction in total viral yield (19, 42). Together, these results underscore that although HA and NA mediate protection through distinct mechanisms, NA-targeted Ad vaccines can elicit a functionally relevant humoral response that warrants further investigation regarding its contribution to overall protection.

Cellular immunity represents another key component of Ad-vectored vaccine protection. We found that HAd-H7HA elicited robust CD4^+^ T-cell responses in both systemic (spleen) and local (MLN and lung) compartments and a comparable enhancement also was observed in HAd-N9NA group. In contrast, CD8^+^ T-cell activation differed markedly between the two vaccines, with HAd-H7HA inducing strong CD8^+^ responses in all tissues examined, whereas HAd-N9NA generated CD8^+^ T-cell responses primarily within the MLN. This discrepancy may be explained by differences in the repertoire of MHC-I–restricted epitopes encoded by each antigen, as HA contains multiple immunodominant CD8^+^ T-cell epitopes in murine models (43, 44). The enrichment of H7N9 virus-specific CD8^+^ T cells in the MLN suggests preferential priming within the respiratory draining lymphoid tissue, potentially enabling rapid responses upon respiratory viral challenge. It is important to note, however, that the most broadly conserved CD8^+^ T-cell epitopes reside in NP and M1, which remain the strongest targets for cross-reactive cellular immunity (45, 46).

A striking observation in our study was the basal cytokine production and partial protection (60% survival) observed in the HAd-ΔE1E3 empty-vector control group. This finding aligns with our previous work demonstrating that Ad vectors activate innate lymphoid cells (ILC), establishing a form of “trained immunity” (34). ILC activation can promote nonspecific CD4^+^ and CD8^+^ T-cell responses and modulate inflammatory signaling, ultimately conferring partial antigen-independent protection. This innate stimulation likely contributed to delayed morbidity and reduced weight loss in the control animals and highlights the intrinsic adjuvant-like properties of Ad platforms.

Our results showed the heterosubtypic protection is limited. NA subtypes segregate into two major phylogenetic groups as Group 1 (including N1) and Group 2 (including N2 and N9) (12, 47). This phylogenetic structure explains the partial cross-protection (60% survival) observed against H3N2 (Group 2) following HAd-N9NA vaccination, as well as the absence of protection against H1N1 (Group 1). This is further supported by the fact that the overall pairwise sequence identities (**Table S1**) alone cannot account for this protective discrepancy. Instead, the evolutionary divergence between groups appears to exceed the threshold required for effective cross-protective antibody or T-cell recognition. These findings reinforce the concept that a broad protective NA-based vaccine will likely require a multivalent or mosaic design incorporating NA antigens from both phylogenetic groups.

A major innovation of this study was the direct comparison between HAdV-5 and BAd-3 platforms. Building on previous work, BAd offers several advantages including bypassing pre-existing anti-HAd immunity (48), utilizing sialic acid receptors rather than CAR for entry (49), and displaying improved biodistribution and persistence, where it efficiently transduces lung, heart, and kidney tissues in addition to the liver and spleen (50). Furthermore, our prior research established that the BAd platform boosts innate immunity and triggers significantly higher cell-mediated immune responses than HAd vectors, enabling a dose sparing effect for influenza vaccines (25). In addition, i.n. delivery of these Ad vectors elicits significantly enhanced mucosal IgA and IgG responses in the respiratory tract compared to systemic routes, directly correlating with superior protection against respiratory pathogens (25). Consistent with these properties, BAd-N9NA elicited stronger mucosal humoral and systemic cellular immune responses than its HAd counterpart. Nonetheless, these immunological advantages did not translate into improved heterosubtypic protection against H1N1, reinforcing that the breadth of protection is constrained by antigenic similarity rather than vector potency.

It should be noted that the HAd and BAd platforms were evaluated using different H7N9 viruses and doses based on the distinct virological properties of their backbones. While the HAd platform was tested against 5 MLD_50_ of the wild-type, non-mouse-adapted A/Anhui/1/2013 isolate, the BAd platform was subjected to a more stringent challenge consisting of 10 MLD_50_ of the pathogenic, mouse-adapted PR8-backed A/Shanghai/2/2013 virus. Although this discrepancy in challenge viruses and stringency limits a direct, quantitative head-to-head comparison between the two vectors, the BAd platform’s ability to elicit robust immunogenicity and complete protection under a significantly harsher challenge environment highlights its potent developmental potential. The highly significant differences in survival and body weight retention compared to their respective mock controls demonstrate that both platforms independently confer robust protective efficacy against H7N9 infection. Nevertheless, a limitation of this study is that post-challenge lung viral titers were not determined. While our survival and body weight data clearly demonstrate robust protection, future studies measuring viral loads will be necessary to confirm whether these vaccines limit viral replication kinetics or achieve complete viral clearance in the lungs.

It is worth noting that intravascular perfusion was not performed prior to lung harvest in the current protocol, meaning that circulating intravascular lymphocytes may partially contribute to the observed pulmonary T-cell profiles. To strictly delineate tissue-resident memory T cells (T_RM_) from circulating contamination, future applications utilizing intravascular antibody staining coupled with established T_RM_ markers (51, 52) are highly warranted. Additionally, while our current cellular cytokine profiles and robust antibody isotype data consistently underscore a well-balanced T_H_1/T_H_2 immune polarization, explicitly expanding future panels to track other auxiliary helper lineages like T_H_2 (IL-5) and T_H_17 (IL-17) will provide more granular mechanistic details. Future comprehensive investigations specializing in the quantification of germinal center (GC) B cells, T follicular helper (Tfh) cells, and localized microenvironmental responses within the nasal-associated lymphoid tissue (NALT) will also be crucial to fully elucidate and optimize these i.n. Ad vaccine platforms. Finally, while the use of inactivated whole virus for ex vivo stimulation provided a robust physiological readout of recall immunity, it requires active antigen processing and may underrepresent the less abundant NA epitopes within the brief stimulation window. Future optimization utilizing synthetic NA-derived peptide pools or specialized MHC tetramers will be highly valuable to directly characterize and quantify epitope-specific T-cell receptor kinetics without the confounding factors of whole-pathogen dynamics. In addition, while specialized functional assays such as Enzyme-Linked Lectin Assay (ELLA), ADCC, and cross-binding panels were not evaluated in this initial study, the robust *in vivo* protection and antibody profiles observed likely reflect these non-neutralizing effector mechanisms. Detailed characterization of these antibody qualities will be important in future studies to fully delineate the fine mechanisms of NA-mediated protection.

In conclusion, our findings demonstrate that Ad-vectored NA vaccines provide robust homosubtypic protection by generating a synergistic combination of mucosal IgA, systemic IgG, and strong CD4^+^ T-cell responses. The BAd platform further amplifies these responses and represents a promising delivery system for mucosal respiratory vaccines. Importantly, The N9NA Ad vaccination provides robust homosubtypic protection but limited within-group heterosubtypic protection. Moving forward, while NA-expressing Ad vectors excel at promoting homosubtypic immunity, their limited within-group heterosubtypic protection underscores that they are best utilized as a promising component within multivalent or multi-antigen influenza vaccine strategies. Future studies should explore combining NA with other conserved targets such as M2e or NP, or co-delivering HA and NA within a unified platform to establish multilayered protection that is less vulnerable to antigenic drift in any single viral protein.

## Data Availability

The original contributions presented in the study are included in the article/**Supplementary Material**. Further inquiries can be directed to the corresponding authors.

## References

[1] Peacock TP Moncla L Dudas G VanInsberghe D Sukhova K Lloyd-Smith JO . The global H5N1 influenza panzootic in mammals. Nature. (2025) 637:304–13. doi: 10.1038/s41586-024-08054-z 39317240

[2] Li Q Zhou L Zhou M Chen Z Li F Wu H . Epidemiology of human infections with avian influenza A(H7N9) virus in China. N Engl J Med. (2014) 370:520–32. doi: 10.1056/nejmoa1304617 23614499 PMC6652192

[3] Zhang J Huang L Liao M Qi W . H9N2 avian influenza viruses: challenges and the way forward. Lancet Microbe. (2023) 4:e70–1. doi: 10.1016/s2666-5247(22)00305-6 36372076

[4] Eisfeld AJ Biswas A Guan L Gu C Maemura T Trifkovic S . Pathogenicity and transmissibility of bovine H5N1 influenza virus. Nature. (2024) 633:426–32. doi: 10.1038/s41586-024-07766-6 38977017 PMC11390473

[5] Neumann G Kawaoka Y . Highly pathogenic H5N1 avian influenza virus outbreak in cattle: the knowns and unknowns. Nat Rev Microbiol. (2024) 22:525–6. doi: 10.1038/s41579-024-01087-1 39060613 PMC12720498

[6] Russell CA Fouchier RAM Ghaswalla P Park Y Vicic N Ananworanich J . Seasonal influenza vaccine performance and the potential benefits of mRNA vaccines. Hum Vaccin Immunother. (2024) 20:2336357. doi: 10.1080/21645515.2024.2336357 38619079 PMC11020595

[7] Wu NC Ellebedy AH . Targeting neuraminidase: the next frontier for broadly protective influenza vaccines. Trends Immunol. (2024) 45:11–9. doi: 10.1016/j.it.2023.11.001 38103991 PMC10841738

[8] Harris A Cardone G Winkler DC Heymann JB Brecher M White JM . Influenza virus pleiomorphy characterized by cryoelectron tomography. Proc Natl Acad Sci USA. (2006) 103:19123–7. doi: 10.1073/pnas.0607614103 17146053 PMC1748186

[9] Gaymard A Le Briand N Frobert E Lina B Escuret V . Functional balance between neuraminidase and haemagglutinin in influenza viruses. Clin Microbiol Infect. (2016) 22:975–83. doi: 10.1016/j.cmi.2016.07.007 27424943

[10] Altman MO Bennink JR Yewdell JW Herrin BR . Lamprey VLRB response to influenza virus supports universal rules of immunogenicity and antigenicity. Elife. (2015) 4. doi: 10.7554/elife.07467 26252514 PMC4552221

[11] Kosik I Yewdell JW . Influenza hemagglutinin and neuraminidase: yin-Yang proteins coevolving to thwart immunity. Viruses. (2019) 11. doi: 10.3390/v11040346 31014029 PMC6520700

[12] Russell RJ Haire LF Stevens DJ Collins PJ Lin YP Blackburn GM . The structure of H5N1 avian influenza neuraminidase suggests new opportunities for drug design. Nature. (2006) 443:45–9. doi: 10.1038/nature05114 16915235

[13] Sandbulte MR Westgeest KB Gao J Xu X Klimov AI Russell CA . Discordant antigenic drift of neuraminidase and hemagglutinin in H1N1 and H3N2 influenza viruses. Proc Natl Acad Sci USA. (2011) 108:20748–53. doi: 10.1073/pnas.1113801108 22143798 PMC3251064

[14] Creytens S Pascha MN Ballegeer M Saelens X de Haan CAM . Influenza neuraminidase characteristics and potential as a vaccine target. Front Immunol. (2021) 12:786617. doi: 10.3389/fimmu.2021.786617 34868073 PMC8635103

[15] Chen YQ Wohlbold TJ Zheng NY Huang M Huang Y Neu KE . Influenza infection in humans induces broadly cross-reactive and protective neuraminidase-reactive antibodies. Cell. (2018) 173:417–429.e10. doi: 10.1016/j.cell.2018.03.030 29625056 PMC5890936

[16] Krammer F Palese P . Advances in the development of influenza virus vaccines. Nat Rev Drug Discov. (2015) 14:167–82. doi: 10.1038/nrd4529 25722244

[17] Freyn AW Ramos da Silva J Rosado VC Bliss CM Pine M Mui BL . A Multi-Targeting, Nucleoside-Modified mRNA Influenza Virus Vaccine Provides Broad Protection in Mice. Mol Ther. (2020) 28:1569–84. doi: 10.1016/j.ymthe.2020.04.018 32359470 PMC7335735

[18] Wohlbold TJ Nachbagauer R Xu H Tan GS Hirsh A Brokstad KA . Vaccination with adjuvanted recombinant neuraminidase induces broad heterologous, but not heterosubtypic, cross-protection against influenza virus infection in mice. mBio. (2015) 6:e02556. doi: 10.1128/mbio.02556-14 25759506 PMC4453582

[19] Krammer F Fouchier RAM Eichelberger MC Webby RJ Shaw-Saliba K Wan H . NAction! How can neuraminidase-based immunity contribute to better influenza virus vaccines? mBio. (2018) 9. doi: 10.1128/mbio.02332-17 29615508 PMC5885027

[20] Kingstad-Bakke B Kamlangdee A Osorio JE . Mucosal administration of raccoonpox virus expressing highly pathogenic avian H5N1 influenza neuraminidase is highly protective against H5N1 and seasonal influenza virus challenge. Vaccine. (2015) 33:5155–62. doi: 10.1016/j.vaccine.2015.08.005 26271828

[21] McMahon M Kirkpatrick E Stadlbauer D Strohmeier S Bouvier NM Krammer F . Mucosal Immunity against Neuraminidase Prevents Influenza B Virus Transmission in Guinea Pigs. mBio. (2019) 10. doi: 10.1128/mbio.00560-19 31113896 PMC6529633

[22] Wang WC Sayedahmed EE Alhashimi M Elkashif A Gairola V Murala MST . Adenoviral vector-based vaccine expressing hemagglutinin stem region with autophagy-inducing peptide confers cross-protection against group 1 and 2 influenza A viruses. Vaccines (Basel). (2025) 13. doi: 10.3390/vaccines13010095 39852874 PMC11769558

[23] Wang WC Sayedahmed EE Alhashimi M Elkashif A Gairola V Murala MST . A bovine adenoviral-vector-based universal influenza vaccine confers protection against influenza A and B viruses in mice and ferrets. Mol Ther Nucleic Acids. (2025) 36:102594. doi: 10.1016/j.omtn.2025.102594 40635672 PMC12240180

[24] Waddington SN McVey JH Bhella D Parker AL Barker K Atoda H . Adenovirus serotype 5 hexon mediates liver gene transfer. Cell. (2008) 132:397–409. doi: 10.1016/j.cell.2008.01.016 18267072

[25] Sayedahmed EE Hassan AO Kumari R Cao W Gangappa S York I . A Bovine Adenoviral Vector-Based H5N1 Influenza -Vaccine Provides Enhanced Immunogenicity and Protection at a Significantly Low Dose. Mol Ther Methods Clin Dev. (2018) 10:210–22. doi: 10.1016/j.omtm.2018.07.007 30101154 PMC6082999

[26] van Olphen AL Mittal SK . Development and characterization of bovine x human hybrid cell lines that efficiently support the replication of both wild-type bovine and human adenoviruses and those with E1 deleted. J Virol. (2002) 76:5882–92. doi: 10.1128/jvi.76.12.5882-5892.2002 12021321 PMC136187

[27] Sayedahmed EE Kumari R Mittal SK . Current use of adenovirus vectors and their production methods. Methods Mol Biol. (2019) 1937:155–75. doi: 10.1007/978-1-4939-9065-8_9 30706395 PMC8761255

[28] Ridenour C Johnson A Winne E Hossain J Mateu-Petit G Balish A . Development of influenza A(H7N9) candidate vaccine viruses with improved hemagglutinin antigen yield in eggs. Influenza Other Respir Viruses. (2015) 9:263–70. doi: 10.1111/irv.12322 25962412 PMC4548996

[29] Alhashimi M Sayedahmed EE Elkashif A Chothe SK Wang WC Murala MST . A multi-antigen-based SARS-CoV-2 vaccine provides higher immune responses and protection against SARS-CoV-2 variants. NPJ Vaccines. (2025) 10:159. doi: 10.1038/s41541-025-01198-7 40683879 PMC12276352

[30] Hassan AO Amen O Sayedahmed EE Vemula SV Amoah S York I . Adenovirus vector-based multi-epitope vaccine provides partial protection against H5, H7, and H9 avian influenza viruses. PloS One. (2017) 12:e0186244. doi: 10.1371/journal.pone.0186244 29023601 PMC5638338

[31] Hoelscher MA Garg S Bangari DS Belser JA Lu X Stephenson I . Development of adenoviral-vector-based pandemic influenza vaccine against antigenically distinct human H5N1 strains in mice. Lancet. (2006) 367:475–81. doi: 10.1016/s0140-6736(06)68076-8 16473124 PMC2762105

[32] DeLano W . Pymol: An open-source molecular graphics tool. 2002. (2002) 40:82–92.

[33] Cao W Liepkalns JS Hassan AO Kamal RP Hofstetter AR Amoah S . A highly immunogenic vaccine against A/H7N9 influenza virus. Vaccine. (2016) 34:744–9. doi: 10.1016/j.vaccine.2015.12.062 26765287 PMC4735641

[34] Mboko WP Wang Y Cao W Sayedahmed EE Mishina M Kumar A . Trained ILCs confer adaptive immunity-independent protection against influenza. J Virol. (2025) 99:e0053225. doi: 10.1128/jvi.00532-25 40757858 PMC12455922

[35] Wang WC Sayedahmed EE Sambhara S Mittal SK . Progress towards the Development of a Universal Influenza Vaccine. Viruses. (2022) 14. doi: 10.3390/v14081684 36016306 PMC9415875

[36] Nachbagauer R Feser J Naficy A Bernstein DI Guptill J Walter EB . A chimeric hemagglutinin-based universal influenza virus vaccine approach induces broad and long-lasting immunity in a randomized, placebo-controlled phase I trial. Nat Med. (2021) 27:106–14. doi: 10.1038/s41591-020-1118-7 33288923

[37] Yassine HM Boyington JC McTamney PM Wei CJ Kanekiyo M Kong WP . Hemagglutinin-stem nanoparticles generate heterosubtypic influenza protection. Nat Med. (2015) 21:1065–70. doi: 10.1038/nm.3927 26301691

[38] Kavishna R Kang TY Vacca M Chua BYL Park HY Tan PS . A single-shot vaccine approach for the universal influenza A vaccine candidate M2e. Proc Natl Acad Sci USA. (2022) 119:e2025607119. doi: 10.1073/pnas.2025607119 35320040 PMC9060463

[39] Johansson BE Kilbourne ED . Dissociation of influenza virus hemagglutinin and neuraminidase eliminates their intravirionic antigenic competition. J Virol. (1993) 67:5721–33. doi: 10.1128/jvi.67.10.5721-5723.1993 8371337 PMC237988

[40] Skehel JJ Wiley DC . Receptor binding and membrane fusion in virus entry: the influenza hemagglutinin. Annu Rev Biochem. (2000) 69:531–69. doi: 10.1146/annurev.biochem.69.1.531 10966468

[41] Couzens L Gao J Westgeest K Sandbulte M Lugovtsev V Fouchier R . An optimized enzyme-linked lectin assay to measure influenza A virus neuraminidase inhibition antibody titers in human sera. J Virol Methods. (2014) 210:7–14. doi: 10.1016/j.jviromet.2014.09.003 25233882

[42] Desheva Y Sychev I Smolonogina T Rekstin A Ilyushina N Lugovtsev V . Anti-neuraminidase antibodies against pandemic A/H1N1 influenza viruses in healthy and influenza-infected individuals. PloS One. (2018) 13:e0196771. doi: 10.1371/journal.pone.0196771 29742168 PMC5942809

[43] Spencer JV Braciale TJ . Incomplete CD8(+) T lymphocyte differentiation as a mechanism for subdominant cytotoxic T lymphocyte responses to a viral antigen. J Exp Med. (2000) 191:1687–98. doi: 10.1084/jem.191.10.1687 10811862 PMC2193146

[44] Muralidharan A Gravel C Duran A Larocque L Li C Zetner A . Identification of immunodominant CD8 epitope in the stalk domain of influenza B viral hemagglutinin. Biochem Biophys Res Commun. (2018) 502:226–31. doi: 10.1016/j.bbrc.2018.05.148 29792863

[45] Quiñones-Parra S Grant E Loh L Nguyen TH Campbell KA Tong SY . Preexisting CD8+ T-cell immunity to the H7N9 influenza A virus varies across ethnicities. Proc Natl Acad Sci USA. (2014) 111:1049–54. doi: 10.1073/pnas.1322229111 24395804 PMC3903243

[46] Kim SH Españo E Padasas BT Son JH Oh J Webby RJ . Influenza virus-derived CD8 T cell epitopes: implications for the development of universal influenza vaccines. Immune Netw. (2024) 24:e19. doi: 10.4110/in.2024.24.e19 38974213 PMC11224667

[47] Vavricka CJ Li Q Wu Y Qi J Wang M Liu Y . Structural and functional analysis of laninamivir and its octanoate prodrug reveals group specific mechanisms for influenza NA inhibition. PloS Pathog. (2011) 7:e1002249. doi: 10.1371/journal.ppat.1002249 22028647 PMC3197600

[48] Singh N Pandey A Jayashankar L Mittal SK . Bovine adenoviral vector-based H5N1 influenza vaccine overcomes exceptionally high levels of pre-existing immunity against human adenovirus. Mol Ther. (2008) 16:965–71. doi: 10.1038/mt.2008.12 18301400 PMC2700267

[49] Li X Bangari DS Sharma A Mittal SK . Bovine adenovirus serotype 3 utilizes sialic acid as a cellular receptor for virus entry. Virology. (2009) 392:162–8. doi: 10.1016/j.virol.2009.06.029 19646729 PMC2743766

[50] Sharma A Bangari DS Tandon M Pandey A HogenEsch H Mittal SK . Comparative analysis of vector biodistribution, persistence and gene expression following intravenous delivery of bovine, porcine and human adenoviral vectors in a mouse model. Virology. (2009) 386:44–54. doi: 10.1016/j.virol.2009.01.008 19211122 PMC2728445

[51] Zhang M Li N He Y Shi T Jie Z . Pulmonary resident memory T cells in respiratory virus infection and their inspiration on therapeutic strategies. Front Immunol. (2022) 13:943331. doi: 10.3389/fimmu.2022.943331 36032142 PMC9412965

[52] Borkner L Curham LM Wilk MM Moran B Mills KHG . IL-17 mediates protective immunity against nasal infection with Bordetella pertussis by mobilizing neutrophils, especially Siglec-F(+) neutrophils. Mucosal Immunol. (2021) 14:1183–202. doi: 10.1038/s41385-021-00407-5 33976385 PMC8379078

