# Supplementary material for: Intranasal adenoviral neuraminidase vaccine induces local and systemic immunity and homosubtypic protection against influenza A viruses
Source: Front Immunol. 2026 Oct 22;17:1911139. doi: 10.3389/fimmu.2026.1911139 (PMC13736095; doi:10.3389/fimmu.2026.1911139)
Supplement: Supplementary file 1 [file DataSheet1.pdf]

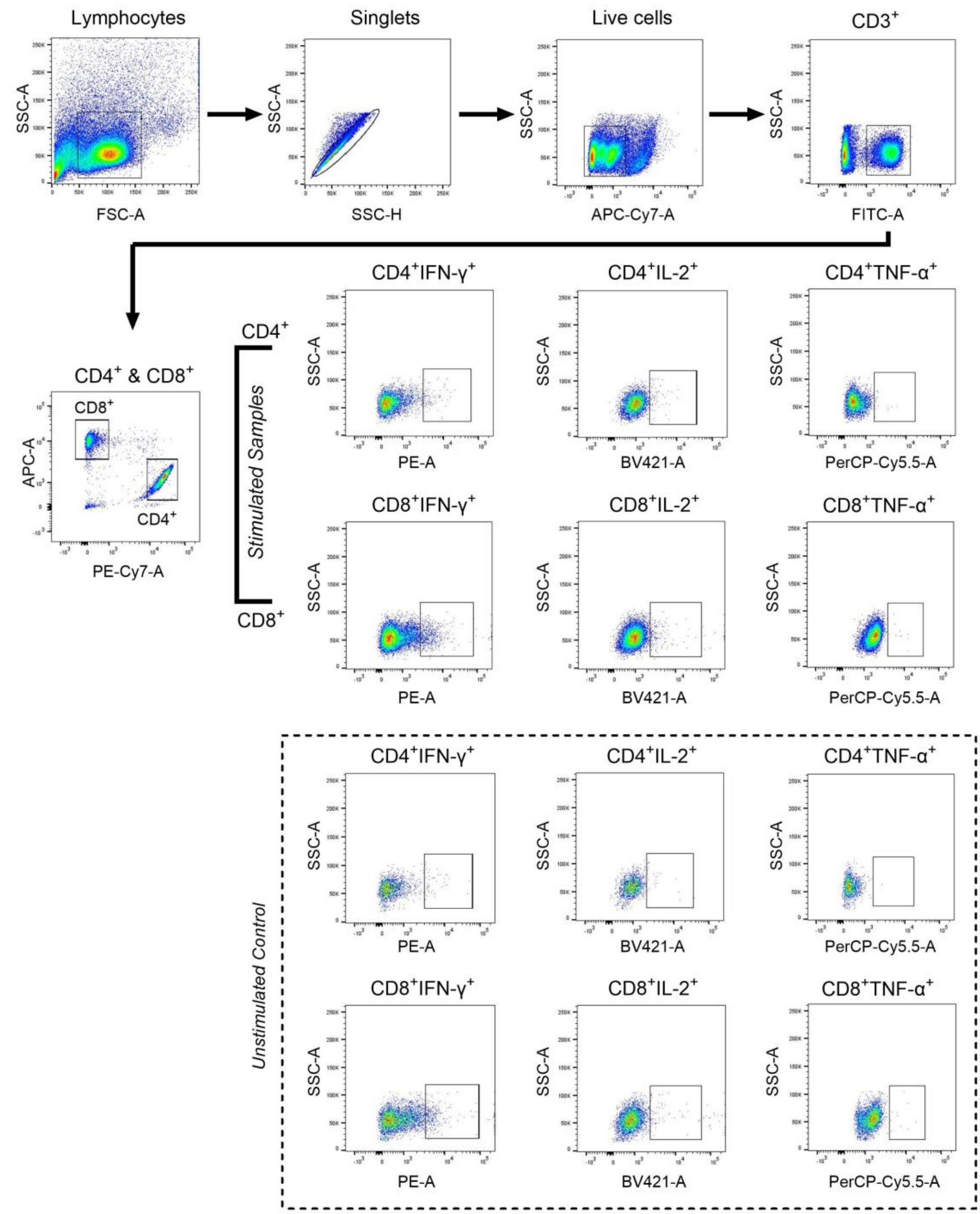

Figure S1. Representative gating strategy for T cell intracellular cytokine staining.

Total lymphocytes were sequentially gated for singlets, live cells, and $CD3^+$ T cells. $CD3^+$ T cells were then divided into $CD4^+$ and $CD8^+$ subpopulations to analyze the expression of IFN-γ, IL-2, and TNF-α. Representative side-by-side plots compare virus-stimulated samples with unstimulated negative controls (dashed box) used to define gating thresholds.

No.	**Vaccine**/challenge strains	1	2	3	4
1	**A/Shanghai/02/2013 (H7N9)**		99.78	45.09	45.53
2	A/Anhui/02/2013 (H7N9)	99.78		45.31	45.75
3	A/PR/8/34 (H1N1)	45.09	45.31		42.86
4	A/HK/01/68 (H3N2)	45.53	45.75	42.86	

created by Clustal2.1

Table S1. Percent amino acid identity between neuraminidase (NA) of influenza A viruses used in vaccine vectors and used for challenges in animal studies.

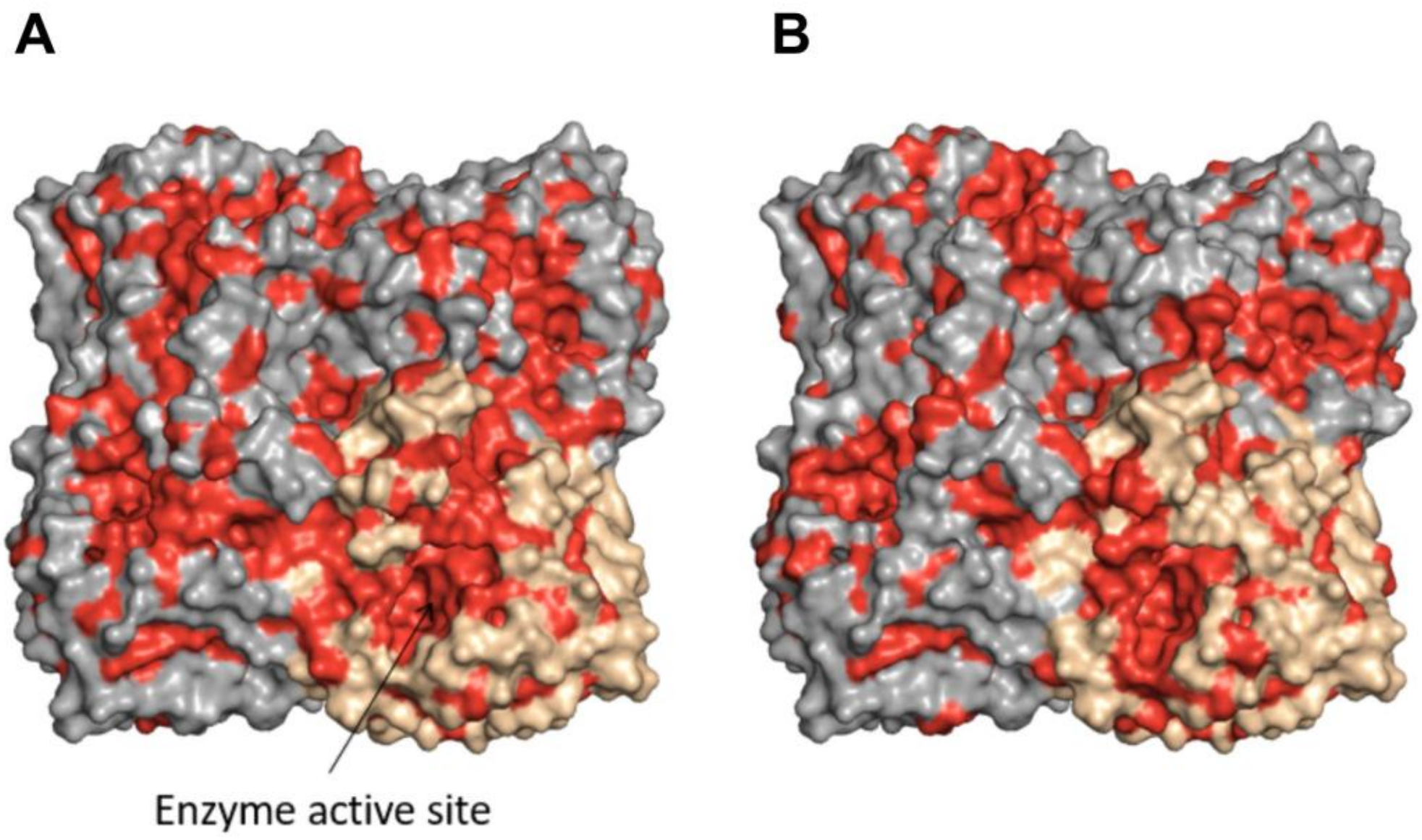

Figure S2. Pairwise structural mapping and surface conservation analysis of the N9 NA tetramer. Structural alignment and surface rendering were generated via PyMOL to compare the spatial distribution of conserved residues. (A) N9 NA surface residues conserved strictly against the N1 NA subtype used in this study are highlighted in red. The arrow indicates the catalytic enzyme active site. (B) N9 NA surface residues conserved strictly against the N2 NA subtype used in this study are highlighted in red. In both panels, a single NA monomer is distinguished in light brown, while non-conserved surface residues are shown in gray.
